# FEVR-Like Presentation of Homocystinuria

**DOI:** 10.1155/2014/646351

**Published:** 2014-11-13

**Authors:** Lorena A. Montalvo, Vincent D. Venincasa, Hassan A. Aziz, Ditte Hess, Audina M. Berrocal

**Affiliations:** Bascom Palmer Eye Institute, University of Miami, 900 NW 17th Street, Miami, FL 33136, USA

## Abstract

A male infant with a diagnosis of homocystinuria presented with avascularity of the peripheral retina with a ridge on ophthalmic exam, consistent with a FEVR-like manifestation homocystinuria. Upon follow-up and treatment for homocystinuria, the retinal vascularity improved without the need for prophylactic treatment to the peripheral avascular retina.

## 1. Introduction

Homocystinuria is a rare disorder caused by a defect in methionine metabolism due to inadequate activity of the enzyme cystathionine beta synthase (CBS) [1]. This results in hyperhomocysteinemia, which is associated with cardiovascular disease (i.e., increased risk of abnormal blood clotting), mild cognitive impairment, and dementia. Ocular manifestations of homocystinuria include ectopia lentis, glaucoma, macular degeneration, maculopathy, retinal degeneration, and retinal vascular diseases, such as central retinal vein occlusion, branch retinal vein occlusion, and central retinal artery occlusion [2].

Familial exudative vitreoretinopathy (FEVR) is a progressive eye disease that affects the vascularization of the peripheral retina [3]. The characteristic manifestations of FEVR include avascularity of the peripheral retina, temporal dragging of the vascular arcades, and heterotopia of the macula. Progression of the disease is indicated with subretinal exudates and tractional retinal detachment [4]. FEVR can be transmitted in a dominant, recessive, or sex-linked fashion and four genes have been implicated thus far [3].

In this unique case report, we describe a male infant affected by a FEVR-like presentation of homocystinuria that resolved with the treatment of the homocystinuria.

## 2. Case Report

A male infant born in Jackson Memorial Hospital at 36 weeks at 2466 grams via cesarean section for premature rupture of membranes was found to have elevated homocysteine levels (101 *μ*mol/L, ref: 4–17 *μ*mol/L) on routine labs. He has no other signs of hypercoagulability and was not given supplemental oxygen. He was referred for genetics evaluation and an ophthalmologic exam at the Bascom Palmer Eye Institute was performed to rule out ectopia lentis.

**Figure 1:**
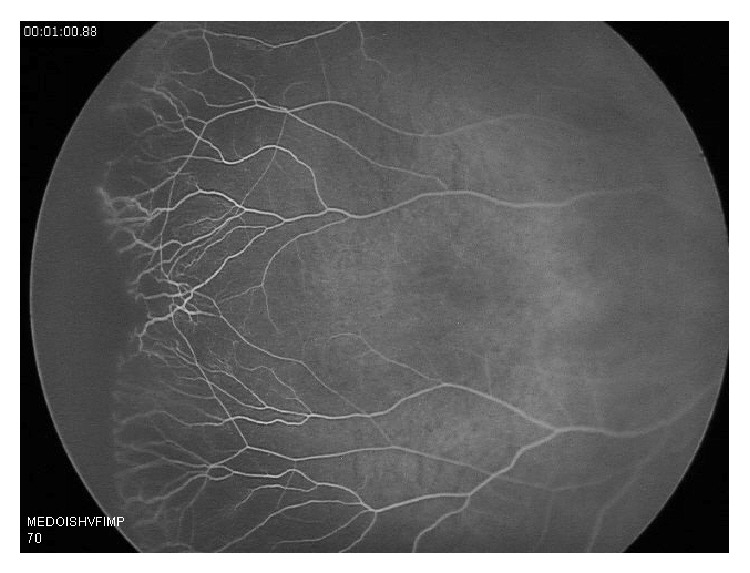
Fluorescein angiogram of the right eye at 1 minute revealing peripheral nonperfusion.

**Figure 2:**
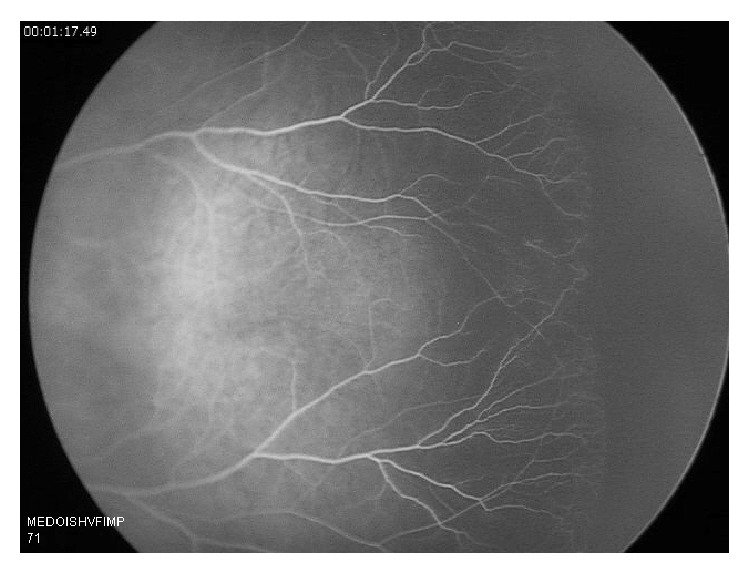
Fluorescein angiogram of the left eye at 1 minute and 17 seconds revealing peripheral nonperfusion.

On exam, the patient appropriately reacted to light, anterior segment exam and conjunctiva showed no abnormality, nonectopic lens and cornea were clear, and the pupils were equally round and reactive. Dilated fundus exam at 1 week (equivalent of 37 weeks gestation) showed grossly attenuated vessels and avascularity of the retina bilaterally. A retinal angiogram was performed and confirmed the diagnosis of avascularity of the peripheral retina, in a FEVR-like distribution (Figures 1 and 2).

Genetic testing showed a heterogeneous unclassified novel missense variant of the cystathionine beta-synthase (CBS) gene, confirming the diagnosis. The patient was then treated with a methionine-free diet, vitamin B6, folic acid, and betaine powder. Upon observation, fundus photographs showed regression of the peripheral ridge, and it was noticed that the retina vessels grew from zone 2 to zone 3 forming normal vascularization of the retina. In the last follow-up at 2 months, his fundus exam showed a flat retina bilaterally with normal vasculature to the ora serrata.

## 3. Discussion

Homocystinuria is a condition associated with increased risk of primary thrombosis [5]. High levels of homocysteine lead to arteriosclerosis, which is characterized by patchy changes in the arterial wall that lack lipid deposits, thought to be secondary to the repair response after repeated mural thrombi [5, 6]. In addition to frank vascular changes, the cystathionine beta-synthase (CBS) enzyme has been shown to play a role in oxidative stress defense and is expressed throughout the human retina [5, 7].

Dysregulation of vascular growth and maturation is most likely responsible for the FEVR-like retinal manifestation of homocystinuria in this patient. While it is unlikely that treatment of homocystinuria could reverse arterial changes so quickly, prompt treatment may reduce oxidative stress and allow for a return to normal development with proliferation of the retinal vessels. This was evident when the patient showed complete vascularization of the peripheral retina on his final examination.

Although ectopia lentis remains one of the most common ocular abnormalities associated with homocystinuria, a complete ocular exam on patients with homocystinuria should be performed to rule out or diagnose retinal abnormalities. Angiography, including wide-field angiography, can be considered to evaluate retinal abnormalities with a vascular component.
